# Could vaccinating adults against malaria materially reduce adult mortality in high-transmission areas?

**DOI:** 10.1186/s12936-023-04714-z

**Published:** 2023-09-19

**Authors:** Hellen Gelband, Ronald Carshon-Marsh, Rashid Ansumana, Ibrahim Bob Swaray, Arjun Pandey, Ashley Aimone, Isaac Bogoch, John Eikelboom, Prabhat Jha

**Affiliations:** 1 Centre for Global Health Research, Unity Health Toronto, University of Toronto, Toronto, Canada; 2 Dalla Lana School of Public Health, University of Toronto, Toronto, Canada; 3 School of Community Health Sciences, Njala University, Bo, Sierra Leone; 4 Department of Medicine, Temerty Faculty of Medicine, University of Toronto, Toronto, Canada; 5 Divisions of General Internal Medicine and Infectious Diseases, Toronto General Hospital, University Health Network, Toronto, Canada; 6 Department of Medicine, University of Toronto, Toronto, Canada; 7 Population Health Research Institute, Hamilton Health Sciences, McMaster University, Hamilton, ON Canada

**Keywords:** Malaria, Adult mortality, Vaccines

## Abstract

After a period of unprecedented progress against malaria in the 2000s, halving the global disease burden by 2015, gains overall in sub-Saharan Africa have slowed and even reversed in some places, beginning well before the COVID-19 pandemic. The highly effective drugs, treated nets, and diagnostics that fueled the initial progress all face some threats to their effectiveness, and global funding to maintain and increase their use over the long term is not guaranteed. Malaria vaccines are among the most promising new interventions that could accelerate the elimination of malaria. Vaccines are still in early stages of rollout in children, the age group (along with pregnant women) that has been the focus of malaria strategies for a century. At the same time, over the past decade, a case has been made, based largely on evidence from verbal autopsies in at least a few high-transmission areas, that the malaria death rate among adults has been greatly underestimated. Could vaccinating adults help to bring down the adult malaria mortality rate, contribute to reduced transmission, or both? A randomized trial of a malaria vaccine is proposed in Sierra Leone, a highly endemic setting, to shed light on this proposition.

## Background

Malaria maintains an intractable hold on much of sub-Saharan Africa. The World Health Organization’s (WHO) 2022 World Malaria Report [1] tells a sobering story of enormous reductions in malaria mortality since 2000, when the global community began to tackle the disease with renewed vigor. Substantial gains continued for a decade, reversing the previously rising rates of cases and deaths, and halving global mortality rates by 2015. The gains slowed and then plateaued between 2010 and 2015. Some increases in cases and deaths in 2020 and 2021 are attributable to service disruptions caused by the COVID-19 pandemic [1], but the momentum had been lost several years earlier.

During this period, and historically, the emphasis on malaria control has been on young children and pregnant women, considered the most vulnerable populations in high-transmission areas. Those who survive childhood and continue to be exposed to infectious mosquito bites develop a partial immunity that provides some protection against severe illness and death, but not necessarily against reinfection. Such immunity has historically been assumed to persist throughout life as long as one resides in a malaria-endemic region. However, a growing body of evidence suggests, at least in some highly endemic areas, a rising death rate beginning in later middle age, resulting in substantial malaria mortality among adults [2, 3].

This perspective explores the question of whether it is worth considering, and testing, the use of malaria vaccines in adults to materially reduce adult morbidity and mortality and contribute to reducing malaria transmission.

### Progress since 2000

The 2000s marked the beginning of a promising new era in malaria control: artemisinins replaced failing chloroquine, and the concept of co-formulated anti-malarials—mainly artemisinin-based combination therapy (ACT)—was accepted globally [4]; chemoprophylaxis (intermittent preventive therapy, IPT) began to be used in pregnant women [5], infants [6] and more recently in school children; rapid diagnostic tests (RDTs) became widely and cheaply available, promising to reduce the presumptive overdiagnosis of malaria and consequent overuse of anti-malarials; and the preventive potential of insecticide-treated nets (ITNs) and indoor residual spraying (IRS) were recognized and became cornerstones of malaria control programmes. Public- and private-sector campaigns to distribute ITNs made millions available all over Africa.

The cost of providing the full suite of interventions—treatment, prevention, and vector control—would be high, but establishment of The Global Fund to Fight AIDS, Tuberculosis and Malaria in 2002 and The US President’s Malaria Initiative in 2005 began to deliver greater funding than had been available ever before. The Bill & Melinda Gates Foundation entered the picture in 2000, supporting operational research and capacity development (and joined the search for an effective and practical malaria vaccine). Global coordination was provided by the Roll Back Malaria Partnership, launched in 1998 by UN agencies and bringing together a wide range of public and private sector actors. The improved tools, substantial financing, and global resolve fueled optimism that the world was on the verge of controlling one of the great scourges of humankind, based on a comprehensive strategy of physical and chemical prevention, testing, and treatment, continuously until elimination and ultimately, eradication.

**Fig. 1:**
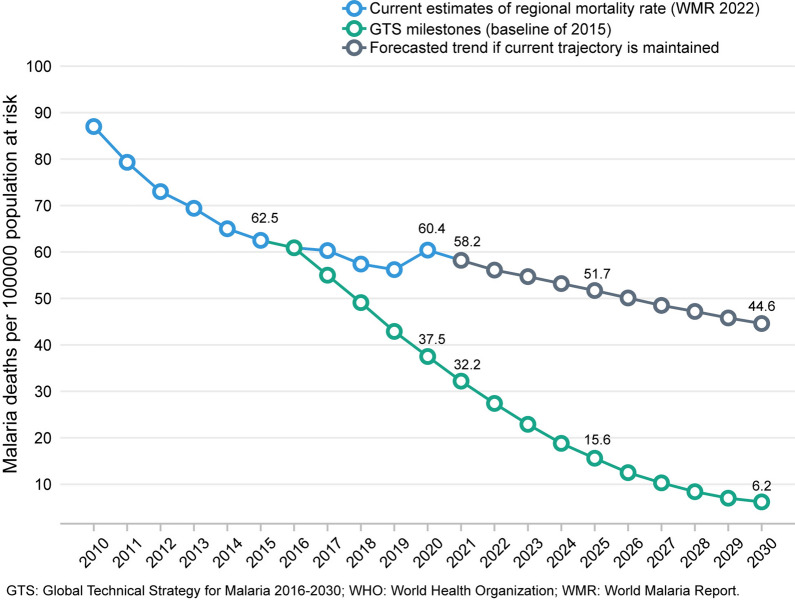
Comparison of progress in malaria mortality rate in the WHO African Region considering two scenarios: current trajectory maintained and GTS targets achieved Source: World Malaria Report 2022 (Fig. 8.4.b)

The result of the expanded and improved efforts was impressive: the estimated worldwide age-standardized malaria mortality rate (driven mostly by childhood deaths) was halved between 2000 and 2015, from 30/100,000 population at risk to 15/100,000. The incidence rate dropped from about 80/1000 at risk to about 60/1000, with the gains concentrated among children [7]. Throughout the period, sub-Saharan Africa bore most of the burden, including 95% of both cases and deaths in 2021. Since 2015, however, the gains have plateaued, especially in sub-Saharan Africa (Fig. 1). While the gains are encouraging, the plateau is too high for malaria to resume meaningful decline under the status quo. Targets set by WHO’s Global Technical Strategy for Malaria 2016–2030 (GTS) [8] have been missed by large margins: the estimated 2021 incidence and mortality rates were twice as high as the targets.

The reasons for lack of continued progress in Africa are likely due to a combination of lower-than-expected effectiveness of the interventions, suboptimal implementation, and insufficient funding [9]. With greater funding and increased effort, the existing tools could be used to greater effect, but the prospect for substantially increased funding and programmatic support cannot be assumed, and the effects might not be large enough to move definitively toward elimination.

In addition, the effectiveness of interventions in current use are threatened by:

1. The rise and spread of artemisinin resistance mainly in Asia, but now seen in African settings [10].
2. Mosquitoes resistant to pyrethroids, the mainstay insecticide used on ITNs [11].
3. The introduction and spread of *Anopheles stephensi*, a mosquito vector that more efficiently transmits malaria in urban environments, is resistant to most insecticides, is an efficient malaria transmitter, and has a high degree of behavioural adaptability [12].
4. False negative rapid diagnostic tests due to histidine-rich protein 2 (HRP2) deletions [13].
5. Substandard and counterfeit drugs and insecticides and inadequate systems of product surveillance.

In addition to these specific threats, the wide and varied current and future effects of climate change loom over malaria control prospects.

Research and development continue for new anti-malarial drugs, insecticides, and strategies, but the only wholly new class of intervention that has become widely available since 2000 are vaccines. The first two approved vaccines, both targeting sporozoites, have become available only recently and are recommended for use in children under 5 years old [14]. Should the potential for these vaccines and others in development—including a promising transmission-blocking vaccine [15]—be more widely investigated in adults to reduce morbidity and mortality, and possibly reduce community transmission, as an added tool for community control and eventual elimination? A randomized trial in adults could also be analysed as a vaccine probe to clarify the contribution of malaria to adult mortality in high-transmission areas [16].

### The case for malaria vaccine trials in adults in high-transmission areas

There is no argument that malaria kills more children than any other age group, but the presumption that malaria mortality remains uniformly low throughout the rest of the lifespan has come increasingly into question. A 2020 perspective piece in *The American Journal of Tropical Medicine and Hygiene* [3] summarized the evidence for a largely unacknowledged burden among adults starting in later middle age in some places with intense transmission. A U-shaped malaria mortality curve, observed initially in India in the first decade of the century [17] was seen also in some African countries during the same time period, as documented by the International Network for the Demographic Evaluation of Populations and Their Health (INDEPTH) network: high mortality in childhood, dropping to low levels by early adulthood, but rising again in middle-age and at older ages. All the cited data were from studies using verbal autopsy (VA) to assign causes of death, which may be subject to misclassifying other infections with similar symptomatology as malaria. The 2020 perspective argued for improved verbal autopsies and confirmatory postmortem biological indicators.

**Fig. 2:**
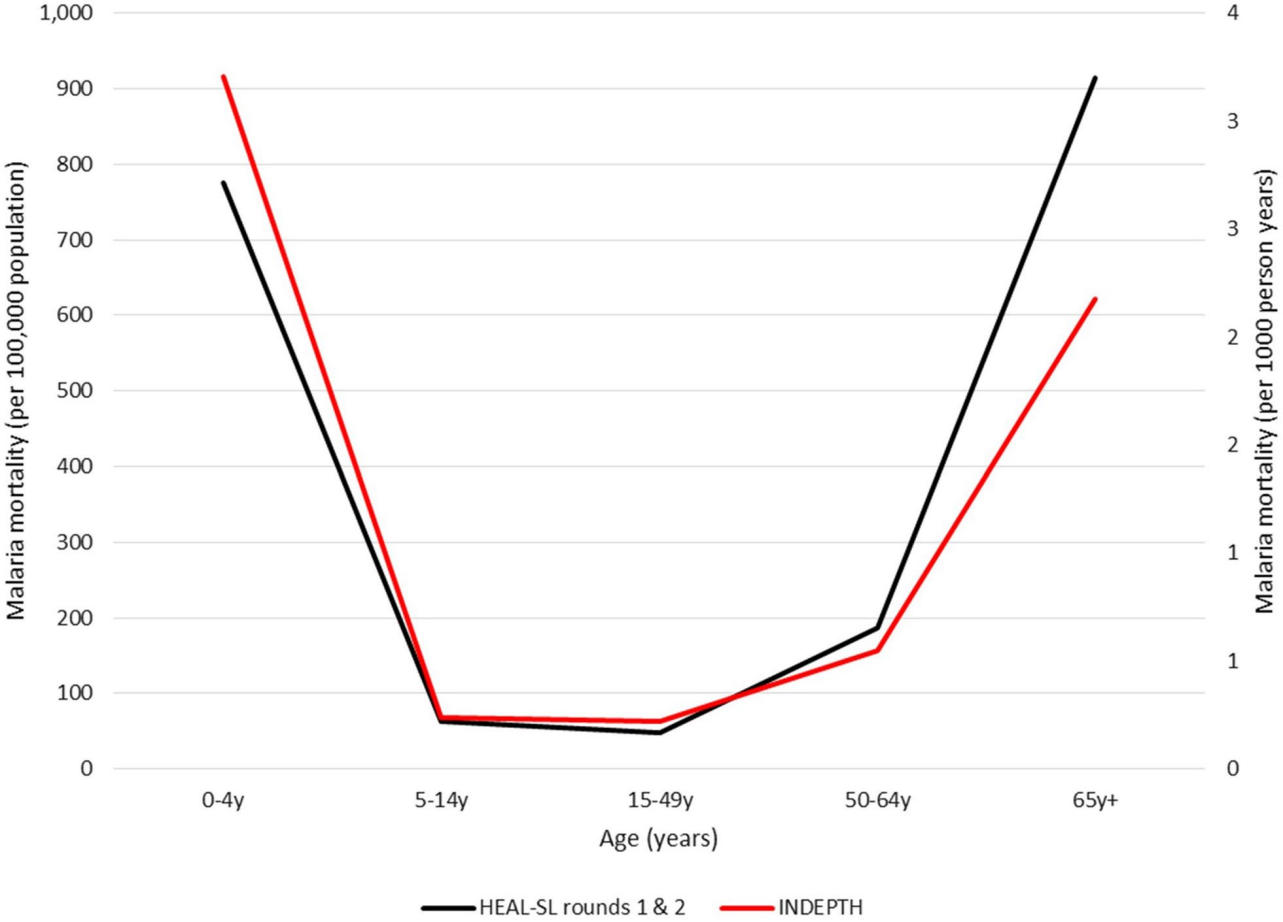
Malaria age-specific death rates in African INDEPTH sites, 2000–2012, and Sierra Leone, 2016–2022 Sources: Gelband et al., [3], Carshon-Marsh et al., [2], and unpublished data from Sierra Leone

Nationally representative cause-of-death reporting for Sierra Leone for the years 2018–2020 was published in 2021 [2], using an improved VA. Malaria was found to be the leading cause of death in all age groups except neonates, with the same U-shaped age-specific death rate pattern seen in the INDEPTH sites (Fig. 2). In all these analyses, if deaths attributed to malaria were misclassified and actually from other causes, a smaller than expected numbers of deaths would be expected in those other categories (e.g., pneumonia, influenza), but this was not apparent. An ongoing study of minimally invasive tissue sampling (MITS) in adults who have died in Sierra Leone should provide additional evidence of whether malaria is an important cause of death, and if it is, of the key clinical signs and symptoms, information that can be used to improve VA questions for the next rounds of surveillance.

In summary, the existing evidence suggests that there may be a substantial risk of adult morbidity and mortality from malaria in high-transmission settings like Sierra Leone, but the evidence can be challenged. With the recent availability of two approved vaccines and others in late-stage trials, a case can be made for field trials in adults with the intention of reducing mortality, and the possible added benefit of reducing transmission to some degree. In addition to the immediate benefits of an effective malaria vaccine, a vaccine probe trial could provide high-quality evidence of the burden of malaria mortality in African adults and inform the accuracy of VA-attributed malaria deaths.

Mass malaria vaccination, rather than vaccinating only children below age 5, has the potential to reduce morbidity and mortality among those vaccinated, both young and old. Under the right conditions, it also may: (1) materially reduce community transmission by reducing the prevalence of circulating gametocytes throughout the population, and (2) reduce the evolution and spread of drug resistance to artemisinins by reducing their use and thus exposure of malaria parasites to them. If vaccination reduces the numbers of infections, the infectious reservoir could be greatly reduced. Individuals with asymptomatic, low-parasitaemic infections, including most infected adults, far outnumber those with symptomatic higher parasitaemias, and as they are unlikely to be treated, their infections may persist for years. Even though they may carry fewer gametocytes per person, they make up a large proportion of the infectious reservoir [18]. Another argument for fielding a trial in adults is to fill in some of the information gaps related to effects of vaccination on transmission by individuals. This could be addressed in detailed laboratory studies of a subsample of participants. The results could inform advanced mathematical modelling of vaccine effects in low- and high-transmission settings.

## Conclusion

The gains of the 2000s in malaria control have plateaued in sub-Saharan Africa, in particular, and are in danger of being eroded because of threats to the effectiveness of the core interventions. At the same time, malaria mortality among adults may be underestimated, especially in high-transmission areas. The recent availability of approved malaria vaccines presents an opportunity to better understand adult malaria mortality and to find out whether use of vaccines beyond children could have a material effect on reducing transmission, with the potential to re-energize malarial control efforts. A large-scale trial of malaria vaccines in adults living in high-burden areas is a first step toward generating this knowledge.

## Data Availability

All data are taken from cited studies except the most recent age-specific mortality data from Sierra Leone, which will be published as quickly as possible, and are available from the corresponding author on reasonable request.

## Abbreviations

ACT: Artemisinin-based combination therapy
COVID: Coronavirus disease
GTS: Global technical strategy for malaria
HEAL-SL: Healthy Sierra Leone
HRP2: Histidine-rich protein 2
INDEPTH: International Network for the Demographic Evaluation of Populations and Their Health
IPT: Intermittent preventive therapy
ITN: Insecticide-treated net
RDT: Rapid diagnostic test
VA: Verbal autopsy
WHO: World Health Organization
